# Evaluating the Clinical Impact of National Dementia Behaviour Support Programs on Neuropsychiatric Outcomes in Australia

**DOI:** 10.3389/fpsyt.2021.652254

**Published:** 2021-04-13

**Authors:** Stephen Macfarlane, Mustafa Atee, Thomas Morris, Daniel Whiting, Madeleine Healy, Marie Alford, Colm Cunningham

**Affiliations:** ^1^The Dementia Centre, HammondCare, St Leonards, NSW, Australia; ^2^Faculty of Medicine, Nursing and Health Sciences, Monash University, Clayton, VIC, Australia; ^3^The Dementia Centre, HammondCare, Wembley, WA, Australia; ^4^Curtin Medical School, Faculty of Health Sciences, Curtin University, Bentley, WA, Australia; ^5^Monash Health, Clayton, VIC, Australia; ^6^School of Public Health and Community Medicine, University of New South Wales, Sydney, NSW, Australia

**Keywords:** DSA model of care, person-centred psychosocial interventions, dementia behaviour support programs, BPSD, neuropsychiatric symptoms, dementia, caregiver distress, retrospective pre-post study

## Abstract

**Background/Objective:** People living with dementia (PLWD) in residential aged care homes (RACHs) are frequently prescribed psychotropic medications due to the high prevalence of neuropsychiatric symptoms, also known as behaviours and psychological symptoms of dementia (BPSD). However, the gold standard to support BPSD is using psychosocial/non-pharmacological therapies. This study aims to describe and evaluate services and neuropsychiatric outcomes associated with the provision of psychosocial person-centred care interventions delivered by national multidisciplinary dementia-specific behaviour support programs.

**Methods:** A 2-year retrospective pre-post study with a single-arm analysis was conducted on BPSD referrals received from Australian RACHs to the two Dementia Support Australia (DSA) programs, the Dementia Behaviour Management Advisory Service (DBMAS) and the Severe Behaviour Response Teams (SBRT). Neuropsychiatric outcomes were measured using the Neuropsychiatric Inventory (NPI) total scores and total distress scores. The questionnaire version “NPI-Q” was administered for DBMAS referrals whereas the nursing home version “NPI-NH” was administered for SBRT referrals. Linear mixed effects models were used for analysis, with time, baseline score, age, sex, and case length as predictors. Clinical significance was measured using Cohen's effect size (*d*; ≥0.3), the mean change score (MCS; 3 points for the NPI-Q and 4 points for the NPI-NH) and the mean percent change (MPC; ≥30%) in NPI parameters.

**Results:** A total of 5,914 referrals (55.9% female, age 82.3 ± 8.6 y) from 1,996 RACHs were eligible for analysis. The most common types of dementia were Alzheimer's disease (37.4%) and vascular dementia (11.7%). The average case length in DSA programs was 57.2 ± 26.3 days. The NPI scores were significantly reduced as a result of DSA programs, independent of covariates. There were significant reductions in total NPI scores as a result of the DBMAS (61.4%) and SBRT (74.3%) programs. For NPI distress scores, there were 66.5% and 69.1% reductions from baseline for the DBMAS and SBRT programs, respectively. All metrics (*d*, MCS, MPC) were above the threshold set for determining a clinically significant effect.

**Conclusions:** Multimodal psychosocial interventions delivered by DSA programs are clinically effective as demonstrated by positive referral outcomes, such as improved BPSD and related caregiver distress.

## Introduction

Dementia is a global health priority with significant socioeconomic costs (1). Regardless of dementia subtype, behaviours and psychological symptoms of dementia (BPSD) are common and difficult to support, affecting up to 90% of individuals with the condition (2). Due to escalated and complex care needs, BPSD are one of the primary reasons for residential aged care placement (3). In fact, up to 91% of people living with dementia (PLWD) in Australia will live their final years in supported residential accommodation (4).

In Australia, there are over 200,000 people living in residential aged care homes (RACHs) (5), where cognitive impairment or dementia affects at least 52% of residents and up to 90% of those are residing in high-level care (6). High quality care involves a person-centred approach, which requires a thorough understanding of the care needs of residents, including those with BPSD. It has been estimated that 10–15% of aged care beds are required to meet the needs of residents exhibiting moderate-to-severe BPSD (7).

BPSD are a complex, heterogeneous and multidimensional constellation of symptoms that may disrupt thoughts, emotions and behaviours (8). BPSD are multifactorial and challenging in nature representing an interplay between biopsychosocial and environmental variables (8). BPSD may result in poor and costly health and care outcomes, such as inappropriate psychotropic prescribing (e.g., antipsychotics) and associated adverse effects (e.g., falls, death), caregiver distress, and hospitalisation (9–12).

Globally, there is an over-reliance on psychopharmacotherapy for BPSD, particularly after entry to RACHs (12–14). In Australia, the scope of this large problem has been highlighted by the Royal Commission into Aged Care Quality and Safety (15). Psychotropic medicines such as antipsychotics have a limited role in treating BPSD and therefore should only be reserved as a last resort therapy (16). In contrast, psychosocial or non-pharmacological interventions are first line support strategies for BPSD (8). These require highly specialised care models, and appropriately skilled and dedicated resources that may not be always available through mainstream aged care services (17). International examples of such care models include the Kansas Bridge Project in the US and the Care Home In-reach program (CHIP) in the UK (18, 19). However, studies of the efficacy of multidisciplinary-led, individualised and nationally consistent behavioural interventions embedded in a mobile care model for the management of BPSD are still scarce.

Person-centred care is one of the key pillars of successful implementation of psychosocial interventions (17). In Australia, a national service provider known as “Dementia Support Australia (DSA)” supports PLWD (referrals) and their caregivers in various care settings through offering multi-disciplinary and multimodal person-centred psychosocial interventions-based programs for BPSD. We hypothesise that DSA programs would have beneficial effects on BPSD for PLWD, including fewer and less severe BPSD episodes, and a decrease in caregiver distress. This study aims to (1) describe and compare the structure and characteristics of DSA programs, and (2) evaluate the impact of these programs on referrals with BPSD from RACHs in terms of improvement in neuropsychiatric or BPSD outcomes for the people supported by the services, and the distress caused to their caregivers (i.e., aged care staff).

## Materials and Methods

### Study Design, Setting, and Population

Outcome data were obtained using a retrospective quasi-experimental single-arm (pre-post) within-subject study design for DSA referrals received during the period 1 January 2018 – 31 December 2019. Established in 2016, DSA is an Australian government funded dementia-specific service that provides individualised psychosocial interventions for people living with mild-to-severe BPSD in Australia.

### Intervention: DSA Programs

#### Description of DSA Programs

**Figure 1:**
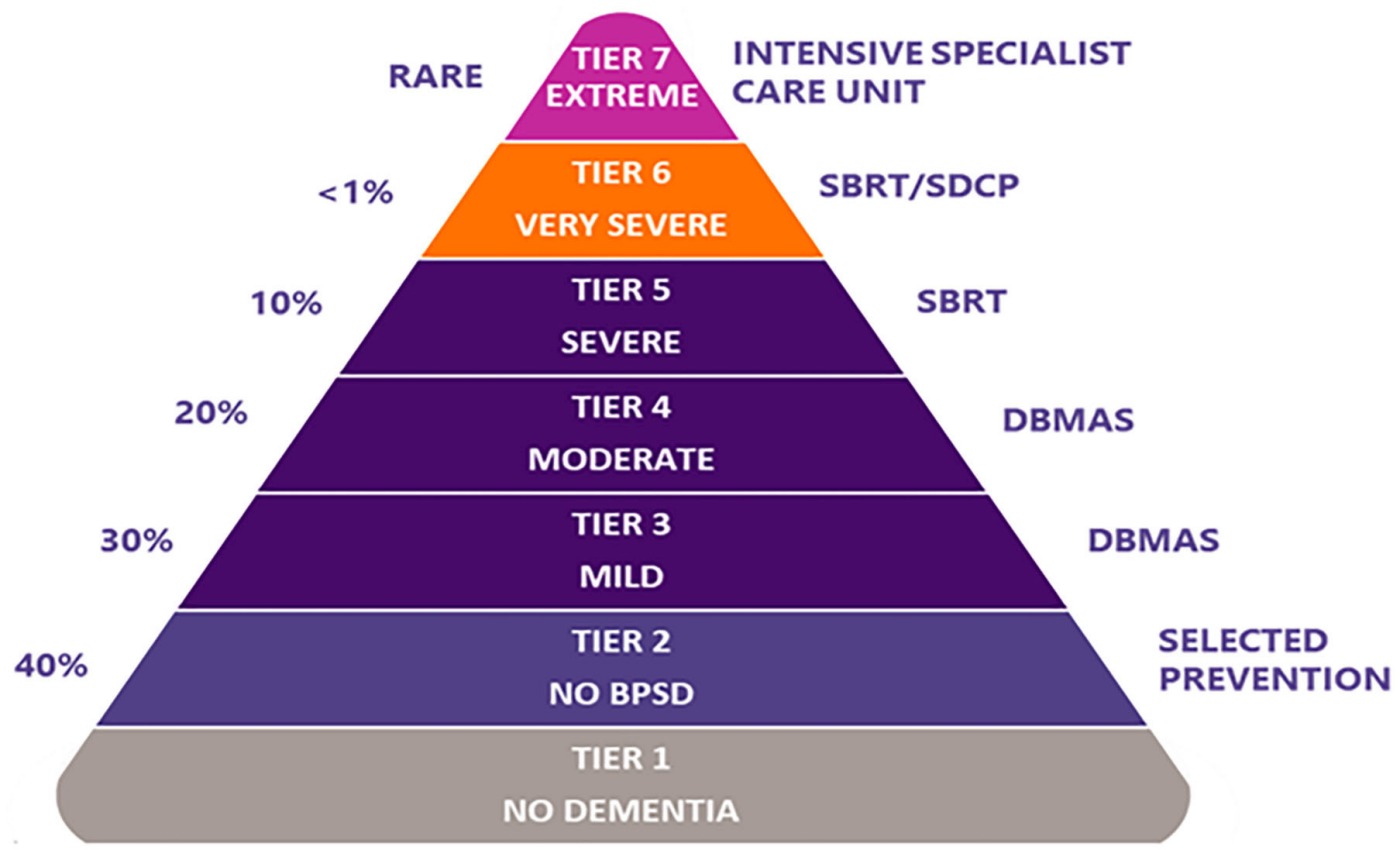
The seven-tiered dementia care model of service delivery, as per Brodaty et al. (20). % on the left represents estimated prevalence for each corresponding tier. BPSD, behaviours and psychological symptoms of dementia; DBMAS, Dementia Behaviour Management Advisory Service; SBRT, Severe Behaviour Response Teams; SDCP, Specialist Dementia Care Program.

DSA programs (Table 1) include the Dementia Behaviour Management Advisory Service (DBMAS) and the Severe Behaviour Response Teams (SBRT). Although both programs have the same model of care and approach (i.e., DSA approach), there are specific and unique characteristics for each (e.g., SBRT mandates a more timely provision of support, and is a dedicated service for those residing in RACHs with greater BPSD severity). BPSD severity, as conceived by DSA, corresponds to the seven-tiered model (Figure 1) proposed by Brodaty et al. (20). This model is commonly referenced within Australia as estimating BPSD severity prevalence and the services required to support BPSD; where tier one represents people with no dementia and no BPSD and tier 7 represents PLWD and extremely severe BPSD. Accordingly, DBMAS supports people within tiers 3 and 4, whereas SBRT covers tiers 5 and 6.

**Table 1:** Key features of “DSA” DBMAS and SBRT programs.

		**DBMAS**	**SBRT**
Program characteristics	Description of the service	1st line of BPSD support; a mobile responsive workforce providing timely expertise and support	2nd line of BPSD support; a mobile responsive workforce available to providing urgent expertise and advice
	Scale of the service	National across all Australian states and territories	
	Length of case management	Shorter-term case management	Longer-term case management
	Client group/Acuity of risks	Mild-to-moderate BPSD	Moderate-to-severe BPSD
	Brodaty Triangle tiers	Tiers 3–4	Tiers 5–6
	Onsite assessment	Within 1-week of referral acceptance	Within 48-h of referral acceptance
	Operational setting	Community and residential aged care	Residential aged care only
	Eligibility criteria	•Have a diagnosis of dementia, or be suspected of having dementia; •Demonstrate evidence of behaviour that impact the care or well-being of PLWD or others; •Demonstrate evidence that the behaviour that warrants referral to DSA is associated with their dementia diagnosis; and •Have consent from the person with dementia or their nominated person responsible for their care	
Program model of care	Philosophy of care	Best practise, multi-modal person-centred care. Collaborative approach (establishing partnerships and relationships to facilitate various clinical and caring arrangements e.g., RACH, local doctors)	
	Interventions	Primarily psychosocial, non-pharmacological and psychoeducational strategies	
	Primary program outcomes	Reduction in BPSD (and related causes e.g., pain) frequency, severity, and related caregiver distress	
	KT activities	Capacity building, and resources for carers and organisations (i.e., referrer)	
	Brokerage	Additional purchased items (e.g., music) and/or support (e.g., external carer) provided in addition to standard DSA services	
Program operating characteristics	Operating processes	A comprehensive assessment of personal history and environment. Support strategies specific for the person are outlined in a written report and discussed with staff or caregivers, with follow up support as needed. CMF, including but not limited to 1. Triage 2. Onsite assessment 3. Recommendation report 4. Ongoing case management and support 5. Eligibility for capacity building and brokerage	
	Mode of service delivery	Telephone, email, videoconference, and onsite/in-person support (including onsite assessment) using CMF	
	Service availability	Access to service is available 24-h a day, 7 days a week	
	Office locations	35 office locations across Australia	
	Staffing	Multi-disciplinary- geriatricians/psychogeriatricians, consultant advisors and capacity building consultants	

*DSA, Dementia Support Australia; DBMAS, Dementia Behaviour Management Advisory Service; SBRT, Severe Behaviour Response Teams; BPSD, behaviours and psychological symptoms of dementia; PLWD, people living with dementia; RACH, residential aged care home; KT, knowledge translation; CMF, case management framework*.

Multi-component psychosocial interventions are routinely applied by DBMAS and SBRT “DSA” consultants using a case management framework (CMF). Through the CMF, consultants work collaboratively with the primary caregivers of a person with dementia and other medical specialists. This involves a thorough assessment of their personal history and surroundings. The primary means by which the consultants support referrals is through providing a recommendation report that outlines factors contributing to behaviours identified during the assessment, and provision of recommendations on how to eliminate or reduce these contributing factors and their impact on BPSD. The DSA philosophy embraces the use of non-pharmacological person-centred approaches as a means of supporting people with BPSD and emphasises the de-prescribing of inappropriate medications. Thus, in certain cases, a medical review by a geriatrician/psychogeriatrician is warranted as part of DSA programs, which occurs in conjunction with the treating general practitioner.

#### Team Members

The DSA team consists of dementia consultants, geriatricians/psychogeriatricians and support staff. DSA consultants are accredited against an industry-specific program focused on dementia-specific competencies and capabilities. Consultants comprise multidisciplinary team members which include, but are not limited to, registered and mental health nurses, occupational therapists, physiotherapists, social workers, psychologists, dieticians, speech pathologists, and diversional therapists with significant experience working in dementia and aged care settings.

#### Eligibility Criteria for DSA Programs

Individuals with a confirmed or probable diagnosis of dementia who have BPSD that impact their care, well-being, or their caregivers.

### Evaluation Protocol

#### Data Source

Primary data was aggregated on a secured database by trained DSA staff after being collected via phone or in-person as part of standard DSA service provision. The data included health, medical and socio-demographic information of referrals. The DSA database is dedicated for documenting and managing case referrals, quality assurance and for follow-up purposes. Each referral was allocated a unique case ID to be tracked across the service. To enhance the delivery of DSA services, data are routinely pooled to measure outcomes and characteristics of those supported by these services.

#### Data Extraction

De-identified records of referrals for a 2-year period (1 January 2018 – 31 December 2019) were extracted from the DSA database by a member of the research team. Data included demographic characteristics (e.g., age), dementia subtypes [e.g., Alzheimer's disease (AD)], Neuropsychiatric Inventory (NPI) scores, and information about the service provision (e.g., intake/discharge dates, NPI administrator, and discharge reasons).

#### Inclusion and Exclusion Criteria for the Analysis

Participants were eligible for the analysis if they met the following criteria: (1) referred to DSA services under DBMAS or SBRT for the study period; (2) resided in RACHs; and (3) had a complete NPI assessment at intake (pre-intervention).

**Figure 2:**
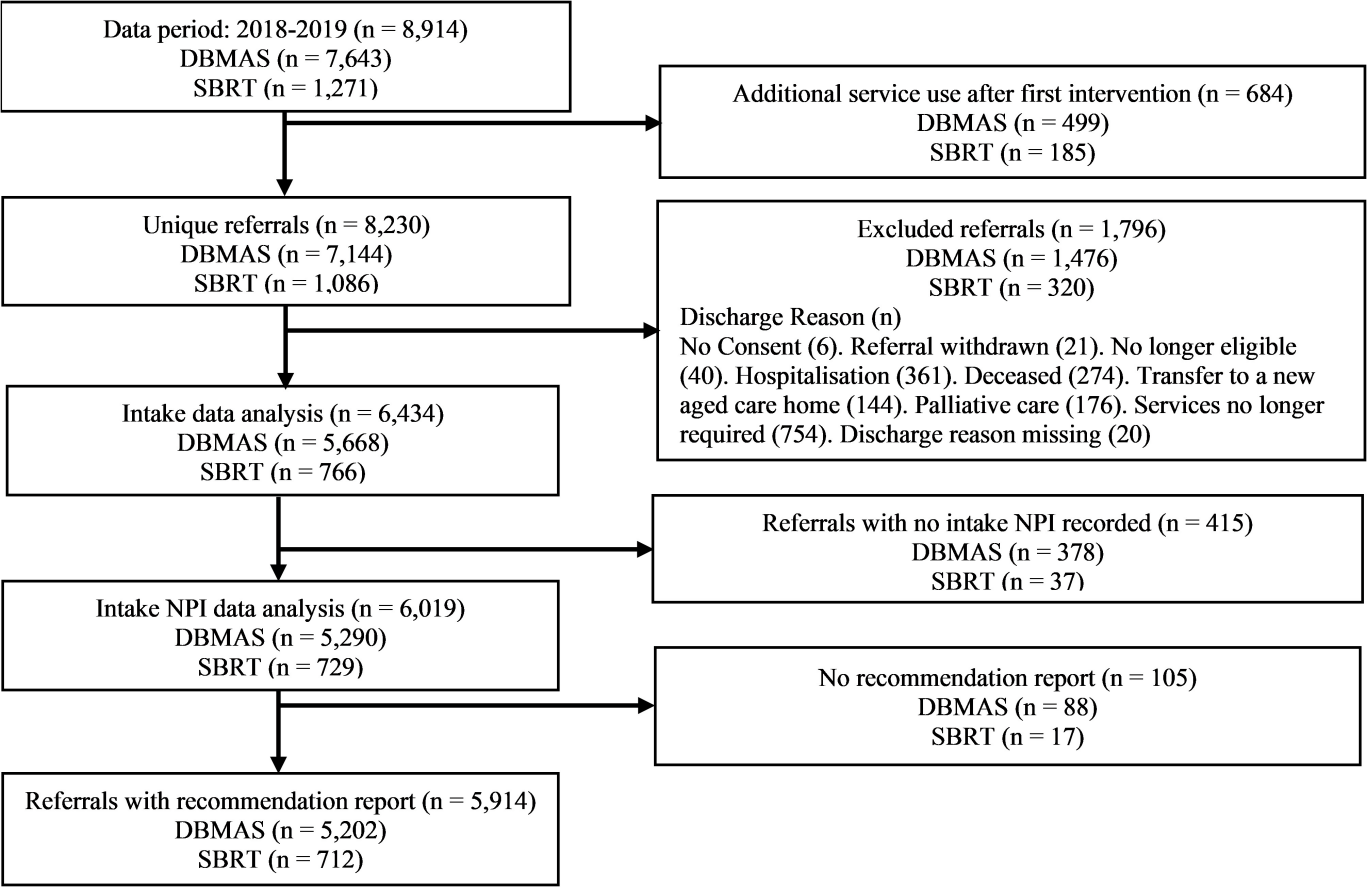
Study design and sample selection flowchart. DBMAS, Dementia Behaviour Management Advisory Service; SBRT, Severe Behaviour Response Teams.

Referrals were excluded from the analysis if no recommendation report could be provided (e.g., consent was withdrawn) or if the discharge reason indicated that the service was unable to be provided (e.g., the person died). If a person was supported by either program multiple times in the study period, only the first instance of service use was included (see Figure 2 for study design and participants flow chart).

For transparency and ease of review, we used the revised SQUIRE 2.0 Statement Checklist to guide our reporting (21).

#### Outcome Measures

The primary outcome measures were BPSD and distress caused to caregivers. BPSD was assessed at intake (baseline) and discharge (endpoint) using two versions of the Neuropsychiatric Inventory (NPI). The questionnaire (shorter) version “NPI-Q” was administered for DBMAS referrals whereas the nursing home version “NPI-NH” was administered for SBRT referrals. Both versions of NPI are informant-based and psychometrically sound instruments (22, 23), which are routinely administered by DSA programs. The NPIs identify the presence (Yes/No) of 12 neuropsychiatric symptoms observed in PLWD, including: aberrant motor behaviour, aggression and agitation, anxiety, apathy, appetite and eating behaviour, delusions, depression, disinhibition, euphoria, hallucinations, irritability, and night-time behaviours. For each domain, a score is given for severity, distress, and for the NPI-NH, frequency. Severity is rated from 1 (mild) to 3 (severe) whereas distress from 0 (not distressing at all) to 5 (extreme or very severe). Frequency in the NPI-NH is rated from 1 (rarely) to 4 (very often). Both versions produce several equivalent domains of overall behaviour, including the total number of behaviours (0–12), the total severity of these behaviours (0–36), and the total distress these behaviours cause caregivers (0–60). For the DBMAS program, the total NPI score is calculated as the sum of the 12 domain severity scores, and for the SBRT program the total score is calculated as the sum of the frequency multiplied by severity scores for each domain (0–144). The total distress score for both the NPI-Q and NPI-NH is calculated as the sum of the distress scores for each domain. Higher NPI scores indicate the presence of more severe or distressing BPSD (22, 23).

#### Data Cleaning and Processing

For the purpose of analysis, rare, or inadequately described dementia subtypes (dementia due to other substance abuse, dementia due to human immunodeficiency virus, dementia associated with Huntington's disease) were collapsed into a single category “other dementia”.

NPI assessment data were screened for duplicates and missing values. Duplicate records were removed. NPI-NH assessment records were considered incomplete if any of the NPI domains of an assessment did not contain scores for both severity and frequency. NPI-Q assessment records were considered incomplete if any of the NPI domains of an assessment did not contain scores for both distress and severity.

#### Statistical Analyses

Descriptive statistics [e.g., count (n), mean, standard deviation (SD), median, percentages] were used to report the demographics of the sample. Differences in demographic characteristics between the programs were evaluated using Welch's *t*-tests and Pearson's Chi-Squared tests for continuous and categorical data, respectively.

We used linear mixed effects (LME) models with total NPI and total NPI distress scores as the dependent variables to evaluate the changes in BPSD from intake to discharge. Each LME model included the following covariates as fixed effects: time (intake/discharge), baseline score, age, sex, and case length. Cases with sex-other were excluded from the LME model analyses. Each model had NPI administrator (i.e., consultant) as a random effect. Despite LME models providing unbiased estimates in the presence of missing data (24), multiple imputation analyses were conducted to ensure no discrepancies in these estimates.

We measured statistical significance using *p*-values and 95% confidence intervals (CIs), while clinical impact (significance) was considered for the difference between NPI intake and discharge scores (total score, total distress) against the following thresholds:

1. Cohen's effect size [$d\;=\frac{Mean\;Intake\;Score\;-\;Mean\;Discharge\;Score\;}{SD_{pooled}}$; ≥0.3]
2. where $SD_{pooled}=\sqrt{\frac{s_{1}^{2}\left(n_{1}-1\right)+s_{2}^{2}\left(n_{2}-1\right)}{n_{1}+n_{2}-2}}$, *n* is the sample size and S^2^ is variance;
3. Mean change score [*MCS* = *Mean Intake Score*−*Mean Discharge Score*; 3 points for the NPI-Q and 4 points for the NPI-NH]; and
4. Mean percent change [$MPC=\frac{MCS}{Mean\;Intake\;Score}$; ≥30%] (25–27).

Descriptive statistics for changes in distress and total scores for individual domains and by dementia subtypes were examined and tabulated to support interpretation of the primary results from the LME models. Two additional LME models for total severity and distress scores were run to obtain the change scores for each dementia subtype, controlling for age, sex, baseline scores and case length. These models combined the DBMAS and SBRT sample and included referrals with the main dementia subtypes [Alzheimer's disease (AD), mixed dementia (MD), frontotemporal dementia (FTD), dementia with Lewy bodies (DLB), and vascular dementia (VaD)]. These adjusted change scores were then used to calculate the MCS, MPC, and Cohen's *d* for each dementia subtype.

The analyses were conducted using R (version 4.0.3) (28) and the nlme package (version 3.1-150) (29).

## Results

### Sample Demographics

During the 2-year period, 8,914 referrals met the inclusion criteria for the analysis. Of those, 5,914 referrals (55.9% female, age 82.3 ± 8.6 years) remained after applying the exclusions (Figure 2). The sample was drawn from 1,996 Australian RACHs, where over half resided in New South Wales (29.9%) and Victoria (21.5%). The most common types of dementia were Alzheimer's disease (37.4%), vascular dementia (11.7%) and mixed dementia (7.6%). Average case length in DSA programs was 57.2 days (SD = 26.3). In total, referrals were born in 95 countries, with the majority born in Australia (59.3%), the UK (9.6%) and Italy (5.8%), and speaking more than 18 languages, including English (78.6%), Italian (4.6%), Greek (2.7%), Arabic (0.8%) and Spanish (0.5%). SBRT referrals were younger and had a higher proportion of males compared to DBMAS referrals. Demographic data are presented in Table 2.

**Table 2:** Sample demographics.

**Characteristics**	**Overall sample** ***n* = 5,914**	**DBMAS** ***n* = 5,202**	**SBRT** ***n* = 712**	***p*-value**
Age, y				<0.001
Mean (SD)	82.3 (8.6)	82.6 (8.5)	80.1 (8.8)	
Median (IQR)	83.0 (77.0–89.0)	84.0 (78.0–89.0)	81.0 (75.0–86.0)	
Sex, *n* (%)				<0.001
Female	3,304 (55.9)	3,015 (58.0)	289 (40.6)	
Male	2,604 (44.0)	2,182 (42.0)	422 (59.3)	
Other	6 (0.1)	5 (0.1)	1 (0.1)	
Dementia subtype, *n* (%)				0.001
AD	2,212 (37.4)	1,908 (36.7)	304 (42.7)	
DUN	1,894 (32.1)	1,730 (33.2)	164 (23.0)	
VaD	689 (11.7)	596 (11.5)	93 (13.1)	
MD	450 (7.6)	392 (7.5)	58 (8.2)	
DLB	176 (3.0)	153 (2.9)	23 (3.2)	
FTD	200 (3.4)	169 (3.3)	31 (4.4)	
PDD	77 (1.3)	67 (1.3)	10 (1.4)	
ARD	83 (1.4)	71 (1.4)	12 (1.7)	
Other	133 (2.3)	116 (2.2)	17 (2.4)	
Case length, days				0.016
Mean (SD)	57.2 (26.3)	56.8 (25.9)	59.6 (28.7)	
Median (IQR)	52.0 (40.0–69.0)	52.0 (39.0–69.0)	54.0 (40.0–74.0)	
Location, *n* (%)				
New South Wales	1,770 (29.9)	1,586 (30.5)	184 (25.8)	0.005
Victoria	1,269 (21.5)	1,083 (20.8)	186 (26.1)	
Western Australia	672 (11.4)	582 (11.2)	90 (12.6)	
South Australia	777 (13.1)	691 (13.3)	86 (12.1)	
Queensland	1,028 (17.4)	899 (17.3)	129 (18.1)	
Tasmania	213 (3.6)	192 (3.7)	21 (2.9)	
Australian Capital Territory	120 (2.0)	110 (2.1)	10 (1.4)	
Northern Territory	65 (1.1)	59 (1.1)	6 (0.8)	
Primary language, *n* (%)				0.089
English	4,650 (78.6)	4,064 (78.1)	586 (82.3)	
Italian	274 (4.7)	245 (4.7)	29 (4.1)	
Greek	156 (2.6)	140 (2.7)	16 (2.2)	
Croatian	49 (0.8)	45 (0.9)	4 (0.6)	
Arabic	48 (0.8)	45 (0.9)	3 (0.4)	
Spanish	29 (0.5)	27 (0.5)	2 (0.3)	
Cantonese	28 (0.5)	23 (0.4)	5 (0.7)	
Other	329 (5.6)	292 (5.6)	37 (5.2)	
Unknown/missing	351 (5.9)	321 (6.2)	30 (4.2)	
Place of birth by country or region, *n* (%)				0.394
Australia	3,507 (59.3)	3,067 (59.0)	440 (61.8)	
UK	559 (9.4)	483 (9.3)	76 (10.7)	
Italy	341 (5.8)	297 (5.7)	44 (6.2)	
Greece	187 (3.2)	170 (3.3)	17 (2.4)	
China	37 (0.6)	33 (0.6)	4 (0.6)	
Middle East	72 (1.2)	67 (1.3)	5 (0.7)	
India	33 (0.6)	32 (0.6)	1 (0.1)	
Other	807 (13.6)	715 (13.7)	92 (12.9)	
Unknown/missing	371 (6.3)	338 (6.5)	33 (4.6)	

*DBMAS, Dementia Behaviour Management Advisory Service; SBRT, Severe Behaviour Response Teams; SD, standard deviation; IQR, interquartile range; AD, Alzheimer's disease; DUN, dementia unspecified or unknown; VaD, vascular dementia; MD, mixed dementia; DLB, dementia with Lewy bodies; FTD, frontotemporal dementia; PDD, Parkinson's disease dementia; ARD, alcohol-related dementia. Other dementias include dementia in other substance abuse, dementia in human immunodeficiency virus and dementia in Huntington's disease. P-values are from Pearson's Chi-Squared tests*.

### Covariates

There were significant effects of baseline score and case length in all models; however, the standardised coefficients indicate that only baseline scores had a meaningful effect. Sex had a small but significant effect for DBMAS cases, while no significant effect of age was noted in any model. Table 3 shows the relationship between the covariates and the NPI scores for each DSA program.

**Table 3:** Association between total NPI and total distress scores and other covariates for each DSA program.

	**DBMAS**						**SBRT**					
	**B (95% CI)**	**SE**	**ß**	**df**	***t***	***p***	**B (95% CI)**	**SE**	**ß**	**df**	***t***	***p***
**NPI total**	***R***^****2****^ **=** **0.79**						***R***^****2****^ **=** **0.81**					
(Intercept)	2.745 (2.187, 3.303)	0.285	9.76	8007	9.64	<0.001	11.917 (6.708, 17.126)	2.655	40.20	1115	4.49	<0.001
Time	−5.919 (−6.334, −5.504)	0.212	−5.92	8007	−27.95	<0.001	−29.785 (−32.236, −27.334)	1.249	−29.78	1115	−23.84	<0.001
Age	−0.005 (−0.011, 0.001)	0.003	−0.04	8007	−1.60	0.110	−0.024 (−0.083, 0.035)	0.030	−0.21	1115	−0.80	0.423
Sex - Male	−0.185 (−0.29, −0.081)	0.053	−0.19	8007	−3.48	<0.001	−0.093 (−1.166, 0.981)	0.547	−0.09	1115	−0.17	0.866
Baseline	0.744 (0.734, 0.754)	0.005	4.27	8007	142.80	<0.001	0.719 (0.693, 0.744)	0.013	15.41	1115	55.26	<0.001
Case Length	0.003 (0.001, 0.006)	0.001	0.09	8007	3.30	<0.001	0.022 (0.003, 0.04)	0.009	0.62	1115	2.31	0.021
**NPI distress**	***R***^**2**^ **=** **0.80**						***R***^**2**^ **=** **0.79**					
(Intercept)	3.62 (2.836, 4.403)	0.400	13.29	8007	9.06	<0.001	5.235 (3.008, 7.462)	1.135	17.82	1115	4.61	<0.001
Time	−8.752 (−9.397, −8.107)	0.330	−8.75	8007	−26.61	<0.001	−12.374 (−13.439, −11.31)	0.542	−12.37	1115	−22.81	<0.001
Age	−0.007 (−0.015, 0.002)	0.004	−0.06	8007	−1.50	0.134	−0.013 (−0.038, 0.012)	0.013	−0.12	1115	−1.04	0.298
Sex - Male	−0.246 (−0.393, −0.098)	0.075	−0.25	8007	−3.27	0.001	0.018 (−0.438, 0.474)	0.232	0.02	1115	0.08	0.938
Baseline	0.745 (0.735, 0.755)	0.005	6.13	8007	146.86	<0.001	0.722 (0.695, 0.749)	0.014	6.15	1115	52.22	<0.001
Case length	0.006 (0.003, 0.009)	0.002	0.16	8007	4.14	<0.001	0.012 (0.004, 0.02)	0.004	0.35	1115	3.07	0.002

*DBMAS, Dementia Behaviour Management Advisory Service; SBRT, Severe Behaviour Response Teams; B, unstandardised coefficient; ß, standardised coefficient; df, degrees of freedom; t, t score value; p, probability value; R^2^, Nakagawa's marginal R^2^ representing the proportion of variance explained by the fixed effects. P-values are from t-tests. Each coefficient represents the effect of each covariate adjusting for the other covariates in the model. For the DBMAS program, the total NPI score is calculated as the sum of the 12 domain severity scores, and for the SBRT program the total score is calculated as the sum of the frequency multiplied by severity scores for each domain (0–144). The total distress score for both the NPI-Q and NPI-NH is calculated as the sum of the distress scores for each domain*.

### Neuropsychiatric Outcomes

Of the referrals eligible for inclusion in the analysis, 2,970 (59%) DBMAS and 502 (71%) SBRT cases had a discharge NPI assessment recorded. For missing data, multiple imputations had no impact on the final conclusions and therefore no imputations were included in any LME model.

Table 4 presents NPI descriptive accounts for each program and the combined sample at intake and discharge.

**Table 4:** Clinical impact of DSA programs on NPI measures.

**NPI measure**	**Combined**		**DBMAS**		**SBRT**	
	**Intake**	**Discharge**	**Intake**	**Discharge**	**Intake**	**Discharge**
Total domains	4.8 (2.2)	2.8 (2.0)	4.7 (2.2)	2.8 (2.0)	5.5 (2.1)	2.9 (2.0)
Total distress	13.7 (8.4)	4.5 (4.8)	13.2 (8.2)	4.3 (4.7)	17.9 (8.4)	5.3 (5.3)
Total severity	10 (5.8)	3.8 (3.5)	9.6 (5.7)	3.7 (3.5)	12.7 (5.6)	4.4 (3.9)
NPI total			9.6 (5.7)	3.7 (3.5)	40.1 (21.3)	10.6 (11.8)
Total frequency					16.9 (7.3)	6.6 (5.6)

*All cells show Mean (SD)*.

*DBMAS, Dementia Behaviour Management Advisory Service; SBRT, Severe Behaviour Response Teams; NPI, Neuropsychiatric Inventory*.

*For the DBMAS program, the total NPI score is calculated as the sum of the 12 domain severity scores, and for the SBRT program the total score is calculated as the sum of the frequency multiplied by severity scores for each domain (0–144). The total distress score for both the NPI-Q and NPI-NH is calculated as the sum of the distress scores for each domain. Lower NPI scores at discharge indicate improvement*.

#### NPI Total Score

There was a significant reduction in total NPI scores for DBMAS (*d* = −1.18, MCS = 5.9, MPC = 61.4%) and SBRT (*d* = −1.66, MCS = 29.8, MPC = 74.3%) from intake to discharge after adjusting for covariates. The predictors in both models explained a large proportion of the variance in NPI scores, with an *R*^2^ of 0.79 and 0.81 for DBMAS and SBRT, respectively (Table 3).

#### NPI Total Distress

There was a significant reduction in total distress scores for DBMAS (*d* = −1.23, MCS = 8.8, MPC = 66.5%) and SBRT (*d* = −1.70, MCS = 12.4, MPC = 69.1%) from intake to discharge after adjusting for covariates. The predictors in both models explained a large proportion of the variance in distress scores, with an *R*^2^ of 0.80 and 0.79 for DBMAS and SBRT, respectively (Table 3).

#### NPI Domains

Individual NPI domains contributed different amounts to the reductions in total scores (see Table 5). For DBMAS, agitation/aggression had the largest reduction (total: *d* = 1.1; distress: *d* = 1.26) while elation had the smallest (total: *d* = 0.15; distress: *d* = 0.14). This was also observed for SBRT with agitation/aggression (total: *d* = 1.93; distress: *d* = 1.94) and elation (total: *d* = 0.16; distress: *d* = 0.18) having the highest and lowest reductions, respectively.

**Table 5:** Clinical impact of DSA programs on NPI domain symptoms.

**Domain**	**Combined**		**DBMAS**		**SBRT**	
	**MPC**	***d***	**MPC**	***d***	**MPC**	***d***
**NPI distress**						
Aberrant motor behaviour	−66.53	−0.59	−66.13	−0.58	−69.40	−0.68
Agitation/aggression	−63.31	−1.29	−63.66	−1.26	−63.71	−1.94
Anxiety	−62.53	−0.71	−61.28	−0.69	−69.80	−0.85
Apathy indifference	−72.69	−0.50	−72.16	−0.50	−75.96	−0.56
Appetite/eating	−77.78	−0.43	−78.58	−0.43	−73.61	−0.42
Delusions	−69.48	−0.49	−68.96	−0.47	−73.36	−0.67
Depression/dysphoria	−67.98	−0.71	−66.62	−0.70	−75.88	−0.82
Disinhibition	−72.98	−0.59	−73.81	−0.57	−71.85	−0.79
Elation/euphoria	−72.15	−0.15	−70.02	−0.14	−83.31	−0.18
Hallucinations	−72.48	−0.34	−71.61	−0.32	−77.42	−0.45
Irritability/lability	−61.57	−0.77	−61.49	−0.73	−64.49	−1.28
Night-time behaviour	−76.65	−0.63	−75.40	−0.60	−83.24	−0.80
**NPI Total**						
Aberrant motor behaviour			−60.08	−0.54	−67.32	−0.65
Agitation/aggression			−55.60	−1.10	−72.69	−1.93
Anxiety			−56.53	−0.65	−72.86	−0.84
Apathy indifference			−67.15	−0.49	−73.20	−0.54
Appetite/eating			−74.81	−0.44	−65.46	−0.37
Delusions			−63.90	−0.44	−77.09	−0.66
Depression/dysphoria			−61.94	−0.69	−76.81	−0.81
Disinhibition			−66.13	−0.51	−72.33	−0.73
Elation/euphoria			−60.99	−0.15	−74.29	−0.16
Hallucinations			−66.17	−0.31	−72.60	−0.40
Irritability/lability			−55.11	−0.65	−74.39	−1.36
Night-time behaviour			−70.99	−0.58	−81.48	−0.73

*DBMAS, Dementia Behaviour Management Advisory Service; SBRT, Severe Behaviour Response Teams; MPC, mean percent change; d, Cohen's effect size*.

*$MPC=\frac{MCS}{Mean\;Intake\;Score}$, where Mean Change Score (MCS) = Mean Intake Score−Mean Discharge Score*.

*No model adjustment used*.

*For the DBMAS program, the total NPI score is calculated as the sum of the 12 domain severity scores, and for the SBRT program the total score is calculated as the sum of the frequency multiplied by severity scores for each domain (0–144). The total distress score for both the NPI-Q and NPI-NH is calculated as the sum of the distress scores for each domain*.

*Negative signs preceding values represent improvement in scores*.

#### Clinical Impact

All metrics (*d*, MCS, MPC) were above the threshold set for determining a clinically significant effect for both programs and both measures (Table 5) and by major dementia subtype (Table 6). In Table 5, the smallest MPC score (61.4%) was well above the 30% threshold. Except for elation/euphoria, all effect sizes were ≥0.3. The MCSs were all above the minimum scores of 3 and 4 for the NPI-Q and NPI-NH, respectively.

**Table 6:** Clinical impact of DSA programs on NPI total severity scores and distress scores by major dementia subtype.

	**Intake**	**Discharge**	**Change**		
**Dementia subtype**	**Mean (SD)**	**Mean (SD)**	**MCS^a^**	**MPC^a^**	***d^a^***
**NPI severity**					
Alzheimer's disease	9.98 (5.89)	3.97 (3.65)	−5.96	−59.71	−1.15
Mixed dementia	9.94 (5.37)	3.44 (3.38)	−6.60	−66.40	−1.39
Frontal lobe dementia	9.99 (5.42)	4.13 (3.39)	−5.94	−59.43	−1.25
Dementia with Lewy bodies	11.85 (6.39)	4.89 (4.79)	−7.09	−59.83	−1.21
Vascular dementia	9.76 (5.71)	3.48 (3.14)	−5.97	−61.17	−1.21
**NPI distress**					
Alzheimer's disease	13.65 (8.47)	4.58 (4.78)	−8.86	−64.90	−1.21
Mixed dementia	13.76 (7.76)	4.41 (5.22)	−9.41	−68.41	−1.36
Frontal lobe dementia	14.02 (8.31)	5.07 (4.91)	−9.06	−64.65	−1.26
Dementia with Lewy bodies	16.13 (9.23)	6.11 (6.42)	−10.30	−63.85	−1.24
Vascular dementia	13.61 (8.47)	4.04 (4.24)	−9.12	−67.01	−1.26

a *MCS, MPC and Cohen's d values represent the change from intake to discharge, controlling for age, sex, baseline score and case length*.

*MCS, mean change score; MPC, mean percent change; d, Cohen's d; M (SD), mean (standard deviation)*.

*Negative signs preceding values represent improvement in scores*.

After adjusting for age, sex, baseline score and case length, the clinical impact of DSA programs on NPI total severity and distress scores exceeded the three clinical threshold values for major dementia subtypes (Table 6). Referrals living with DLB had the greatest MCS improvement while referrals with MD had the greatest MPC and *d* values.

Across major dementias, agitation/aggression had the greatest MCS (range: 0.93–1.14 for total severity; range: 1.61–1.76 for total distress), whereas elation/euphoria had the smallest (range: 0.02–0.09 for total severity; range: 0.03–0.12 for total distress), as presented in Table 7. The highest MPC for total NPI severity scores was recorded for night-time behaviours in FTD, while the lowest was documented for apathy/indifference in DLB. For total distress scores, the highest MPC was noted for elation/euphoria in MD, and the least was seen for hallucinations in DLB when compared with other dementias.

**Table 7:** Clinical impact of DSA programs on NPI total severity scores and distress scores by NPI symptom domain score and dementia subtype.

**Domain**	**AD**			**FTD**			**DLB**			**MD**			**VaD**		
	**Intake**	**MCS**	**MPC**	**Intake**	**MCS**	**MPC**	**Intake**	**MCS**	**MPC**	**Intake**	**MCS**	**MPC**	**Intake**	**MCS**	**MPC**
**Total severity**															
Aberrant motor behaviour	1.03	−0.59	−57.0	1.09	−0.66	−60.3	1.02	−0.63	−61.8	0.83	−0.58	−69.9	0.77	−0.48	−62.4
Agitation/aggression	1.85	−1.01	−54.5	1.84	−0.93	−50.6	1.95	−1.14	−58.5	1.87	−1.11	−59.3	1.88	−1.04	−55.4
Anxiety	1.26	−0.72	−57.1	1.07	−0.56	−52.0	1.39	−0.85	−61.0	1.14	−0.68	−59.9	1.12	−0.68	−61.1
Apathy/indifference	0.63	−0.43	−68.9	0.58	−0.36	−62.2	0.60	−0.26	−43.1	0.67	−0.44	−65.1	0.64	−0.47	−73.4
Appetite and eating	0.45	−0.33	−73.8	0.38	−0.26	−68.1	0.43	−0.21	−48.6	0.38	−0.25	−66.0	0.37	−0.30	−80.0
Delusions	0.62	−0.39	−61.9	0.58	−0.42	−71.7	1.02	−0.58	−56.2	0.63	−0.43	−68.5	0.62	−0.39	−63.1
Depression/dysphoria	1.07	−0.67	−62.6	0.86	−0.54	−62.1	1.22	−0.67	−55.4	1.19	−0.78	−65.9	1.14	−0.78	−68.2
Disinhibition	0.72	−0.44	−61.5	1.32	−0.79	−59.8	0.70	−0.49	−70.0	0.76	−0.56	−73.3	0.80	−0.55	−69.6
Elation/euphoria	0.08	−0.05	−57.9	0.17	−0.09	−53.2	0.09	−0.07	−77.7	0.08	−0.06	−80.3	0.04	−0.02	−44.9
Hallucinations	0.30	−0.19	−65.2	0.25	−0.18	−70.5	1.11	−0.61	−54.7	0.35	−0.26	−74.0	0.27	−0.18	−69.1
Irritability/lability	1.22	−0.67	−55.4	1.21	−0.57	−47.2	1.32	−0.72	−54.5	1.31	−0.77	−59.2	1.39	−0.80	−57.8
Night-time behaviour	0.76	−0.53	−69.7	0.61	−0.50	−82.5	1.00	−0.74	−74.3	0.73	−0.57	−77.8	0.72	−0.57	−78.9
**Total Distress**															
Aberrant motor behaviour	1.40	−0.93	−66.2	1.45	−0.95	−65.4	1.37	−0.84	−61.1	1.16	−0.86	−73.9	1.08	−0.72	−66.2
Agitation/aggression	2.73	−1.70	−62.3	2.83	−1.63	−57.6	2.82	−1.61	−57.1	2.80	−1.76	−63.0	2.79	−1.76	−63.1
Anxiety	1.73	−1.06	−61.5	1.52	−0.84	−54.9	1.92	−1.31	−68.3	1.60	−0.97	−60.7	1.59	−1.11	−69.8
Apathy/indifference	0.74	−0.54	−73.3	0.72	−0.51	−71.3	0.66	−0.37	−56.7	0.82	−0.57	−69.9	0.78	−0.61	−78.8
Appetite and eating	0.53	−0.42	−78.4	0.48	−0.36	−75.8	0.57	−0.37	−65.1	0.48	−0.36	−74.4	0.48	−0.41	−84.9
Delusions	0.86	−0.57	−66.4	0.84	−0.61	−72.7	1.43	−0.77	−53.9	0.89	−0.62	−69.1	0.85	−0.60	−70.4
Depression/dysphoria	1.36	−0.93	−68.0	1.08	−0.68	−63.0	1.51	−0.96	−63.5	1.49	−1.02	−68.4	1.43	−1.07	−74.8
Disinhibition	1.03	−0.72	−70.2	1.98	−1.36	−68.9	0.93	−0.70	−75.3	1.08	−0.84	−78.1	1.14	−0.84	−73.8
Elation/euphoria	0.08	−0.05	−65.2	0.18	−0.12	−64.6	0.10	−0.08	−81.4	0.05	−0.05	−92.2	0.05	−0.03	−62.3
Hallucinations	0.37	−0.27	−72.8	0.32	−0.23	−72.3	1.38	−0.70	−51.0	0.47	−0.34	−72.5	0.36	−0.29	−81.2
Irritability/lability	1.73	−1.05	−60.7	1.73	−0.92	−53.1	1.95	−1.16	−59.4	1.89	−1.13	−60.0	2.03	−1.29	−63.7
Night-time behaviour	1.09	−0.82	−75.7	0.90	−0.75	−83.5	1.49	−1.14	−76.4	1.03	−0.83	−80.7	1.02	−0.83	−81.1

*AD, Alzheimer's disease; FTD, frontotemporal dementia; DLB, dementia with Lewy bodies; MD, mixed dementia; VaD, vascular dementia; MCS, mean change score; MPC, mean percent change*.

*Negative signs preceding values represent improvement in scores*.

The overall impact of DSA programs on each major subtype of dementia was demonstrated by the 12 domain symptoms of the NPI in Table 7. Notably, AD and VaD scored the highest MPC improvement in NPI scores for appetite and eating disorders when compared to other NPI symptoms. In contrast, DLB and MD recorded the greatest MPC improvement for elation/euphoria as opposed to other NPI symptoms. For FTD, the highest MPC value was observed for night-time behaviour.

## Discussion

Person-centred psychosocial interventions are considered a key therapeutic approach for supporting BPSD. DSA programs offer these interventions through national multidisciplinary and individualised care services for people with BPSD. These programs use a CMF to improve and monitor health and care outcomes, such as reducing BPSD and caregiver distress in referrals from various care settings, including those living in RACHs. This study aimed to evaluate the impact of this approach on referrals with BPSD from RACHs, using a pre-post design with a retrospective analysis to investigate the clinical impact of DSA programs on two referral groups with BPSD (i.e., DBMAS for mild-moderate BPSD and SBRT for more severe BPSD).

The key finding was that in both groups the NPI scores were significantly reduced as a result of these programs. The impact of these programs was independent of age, sex, baseline score and case length. A UK study by Smith and Mackenzie found that the Newcastle Challenging Behaviour Team (a 12-week in-reach program) had mean reductions in NPI resident behaviour of 17.92 and of caregiver distress by 5.85 (30). Another program in the US, the Kansas Dementia Crisis Bridge Project was effective in reducing or resolving NPI-Q symptoms (e.g., agitation/aggression score was 1.81 ± 1.11 vs. 0.79 ± 0.64 for pre- and postintervention, respectively) (18). A community mental health team provided a dementia care home in-reach program (CHIP) into 4 RACHs in Birmingham, UK (19). The CHIP project employed person-centred and multidisciplinary non-pharmacological interventions that improved BPSD management in PLWD (19). In their meta-review, Dyer et al. reported an estimated effect size of 0.1–0.49 for non-pharmacological interventions for BPSD (31). Compared with these studies/programs (18, 19, 30), our evaluation was conducted at a national level of implementation and using dementia- and setting-specific tools; hence producing meaningful and representative results. Despite these methodological variations and differing characteristics of these program, our findings are congruent with these earlier studies, but where DSA programs have a much stronger clinical impact and larger (national) reach.

Since 2016, the DSA programs have provided rapid, comprehensive, and intensive dementia-specific in-reach services (e.g., BPSD support and education) across Australia, indicating the feasibility of these programs. In this study, two states (New South Wales and Victoria) represented half of the referrals, as they are the most populous states in Australia (32). The programs responded to PLWD from diverse cultural and linguistic backgrounds representing the multicultural identity of Australian society. This also suggest that DSA services are provided in a culturally and linguistically appropriate manner, which is respectful and mindful of the cultural, linguistic, religious and spiritual preferences, needs or values, or other specific needs such as diet and gender, of people of culturally and linguistically diverse backgrounds. This approach is fundamental to the delivery of person-centred dementia care (PCDC). Such a model of care is important to all individuals living with dementia including those from minority and underrepresented groups. Internationally, geriatric in-reach programs of a similar scope (but with limited feasibility or geographic reach or with no data on ethnocultural identity) include the Dementia Rapid Response Teams (DRRT) (33) and the Dementia and Intensive Support Team (DIST) (34) in the UK, and the Advanced Illness Care Teams (AICT) in the US (35).

Both DSA programs applied a PCDC approach, as articulated by Kitwood's principles (36). Person-centredness involves attributes of compassion, concern, kindness and respect (37), which can be difficult to measure (38). It underscores the value of the individual with their own unique history, experiences, values and culture that have shaped who they are. Previous studies demonstrated that person-centred care in RACHs improved quality of life, and reduced agitation and caregiver burnout (39–41). A recent review outlined that the impact of PCDC was investigated in 8 studies for behavioural disturbances, and 5 studies for emotional disturbances (42). Of those, 5 studies demonstrated significant reductions in agitation and only one study used NPI as a measure of change (42). There are various triggers for BPSD such as staff practises, pain, discomfort, environmental stressors or over stimulation, and thus applying tailored multimodal interventions is the best practise approach (8, 43).

BPSD is an umbrella term for a diverse range of neuropsychiatric (non-cognitive) symptoms that are either instigated by unmet needs (e.g., pain) or linked to psychiatric symptoms (e.g., hallucinations) (43, 44). As such, it is difficult to untangle the impact of the programs unless we specifically focus on individual symptoms or subsyndromes. In our study, all NPI symptoms were responsive to the DSA programs (Tables 5, 7). Although the clinical impact of both programs was meaningful, the SBRT recorded the highest reduction in BPSD. Overall, our data revealed clinically significant reductions in BPSD, and the distress that they cause (Tables 4–7).

The variability in NPI domain reduction scores could have been a result of the difference in the frequency of NPI domains in referrals presented to the DSA programs (i.e., at intake). Agitation/aggression was the most frequent reason for DSA referrals. Further, by large, referrals with agitation/aggression had the highest NPI baseline scores, which was strongly associated with the greatest MCS reduction as a result of DSA programs. Higher baseline scores may respond better to interventions because they have a greater opportunity for improvement compared to other domains, which may have smaller improvements due to floor effects (e.g., for elation which had the lowest baseline scores). This may also indicate that referrals with more severe BPSD might benefit the most from these programs. Additionally, domains with high baseline scores are likely to be the foci of consultants' recommendations, resulting in larger changes in those areas compared to domains that were less severe at the time of referral. Whilst this pattern of change was much clearer for MCSs, it was not the case for MPC scores as these values are calculated differently. Moreover, the observed differences may be at least partially related to symptom fluctuation and interventions applied with time. Other factors that may contribute to score differences are the co-existence of delirium and other medical conditions (e.g., urinary tract infection, pain). This is particularly relevant in PLWD as a large proportion may have multimorbidity and (hyper-)polypharmacy (i.e., receiving 5 or more prescribed medications) (45). Examples of these are psychotropic medications, such as antipsychotics and antidepressants where up to 48% of aged care residents living with dementia receive one or more of these agents; of which over half (54%) have been found to be potentially inappropriate (14, 46).

For each DSA program, a strong association was evident between NPI scores and other covariates, as indicated by the high percentage of explained variance (Table 3) (47). Due to the nature of eligibility and service delivery, there were considerable differences between DBMAS and SBRT referrals in the mean NPI severity and distress scores, sex, and age. Referrals with more severe degrees of BPSD (i.e., those referred to SBRT) demonstrated a greater reduction (74%) in NPI total scores. This may partly be due to floor effects, as DBMAS intake NPI scores were lower than in SBRT, and so had a smaller range of possible reductions. Alternatively, the greater reduction seen in the SBRT cohort may reflect the greater intensity and duration of service provision in that group, enabling them to receive a greater “dose” of the interventions offered.

Regardless of the dementia subtype, the clinical impact of DSA programs was demonstrated for referrals, as noted in Tables 6, 7. While all major dementia subtypes (except for dementia with Lewy bodies “DLB”) had similar baseline scores and subsequent BPSD improvement (as measured by NPI), mixed dementia (MD) had the best response (e.g., MPC values for total severity and distress scores were 66.4 and 68.4%, respectively) to DSA programs. This is perhaps because MD had heterogenous neuropathological aetiologies and clinical features (AD and VaD) with more severe and distressing symptoms, and the unmet needs responsible for these symptoms were easier to recognise and support when compared to other dementias. Further, it is likely that referrals with various types of dementia who were enrolled in DSA programs received different person-centred interventions under variable time frames because they may have been experiencing different symptoms with a variation in severity. These factors, in turn, may have influenced the effectiveness of the programs investigated against different dementia subtypes. Notwithstanding this, it is almost inevitable that the neuropathological trajectories of all dementias eventually progress to a mixed type, where the complexity of symptoms are likely to require and respond to multimodal interventions. Further, from Table 7, it appears to be the characteristic symptom domains of certain dementia subtypes may play a role in the overall response to psychosocial interventions. For example, DLB is highly characterised by psychotic symptoms (delusions and hallucinations) that seem to be less responsive to these interventions. This may be because psychotic symptoms in the context of DLB are more intractable or stable over time (48); and therefore more likely to have an organic aetiology compared to other dementias (which lends further support that these symptoms may respond better to other treatments such as psychotropic medications) (49). In contrast, the highest MPC values in NPI scores for AD and VaD were observed for appetite and eating disorders. However, these differences were offset in our sample by the change in other domain symptoms, making such differences almost negligible in the discharge NPI scores, as noted in Table 6. Finally, the differences in clinical responses to DSA programs may be attributed to the prevalence, persistence or incidence of BPSD profiles in respective dementia subtypes (50).

### Strengths and Limitations

To date, this is the first and largest population-based real-world study that reports neuropsychiatric outcomes in PLWD after program-based psychosocial interventions. A total of 1,996 (out of 2,695; 74.1%) Australian RACHs contributed data to the evaluation, covering a population of PLWD, that is thus highly representative of the Australian residential aged care setting (51). The real-world or pragmatic context for the study allowed DSA consultants to conduct their work without being observed and influenced, eliminating any potential occurrence of the Hawthorne or Observer effect. All DSA consultants involved in delivering the interventions were blinded to the study objectives, methodologies and analyses as the present study had retrospective epidemiological design undertaken by a research group independent to those consultants.

The psychosocial interventions implemented by DSA comprise a multimodal and holistic set of individualised behaviour support strategies. In most instances, these interventions are implemented in parallel with each other. To our knowledge, the interventions offered by DSA represent the first exemplar of a service emphasising these components that has been implemented on both a long-term and a national scale. Despite significant reductions in NPI scores observed over the course of episodes of care, it is not possible, therefore, to describe the nature of the “effective” intervention in anything other than general terms. By examining a large set of data within a 24-month period, we sought to reduce any potential impact of seasonal variation of BPSD. Yet, this may still have had some impact on the results.

Our study is descriptive, retrospective, and employed a single-arm analysis. Given the complexities of the setting, various types of dementia and BPSD, and models of care applied, randomised controlled trials may not be feasible, ethical, or practical. As the data for this study were collected during provision of interventions to PLWD who had been referred to the service for timely assistance and support, it was not possible to use a control group (whether through delaying the delivery of interventions in the instance of own control group or having an independent control group). The lack of control group makes it difficult to rule out effects of regression to the mean, as described by Ballard et al. (52), whereby those with more severe BPSD at intake, and/or those who spend more time in the program would regress towards less severe patterns of behaviour as a result of normal variability. However, given the marked improvements seen at discharge and that the change occurred within a relatively short period of time (57.2 ± 26.3 days), the observed change is likely to be attributed to these programs. Further, our comprehensive analyses were adequate to account for this variability.

The effect sizes reported in the literature vary because of different interventions (e.g., drugs vs. non-drugs) applied or because the sample sizes were too small to calculate the effect size (53). Although other non-pharmacological interventions in the literature have a similar effect size for managing BPSD to the pharmacological interventions, the former have no associated adverse events (31). A recent systematic review of meta-analyses by Tampi et al. found that antipsychotics have only a modest efficacy in treating psychosis, aggression and agitation in PLWD (54). Given the uniqueness of DBMAS and SBRT programs, particularly in applying multimodal person-centred psychosocial/non-pharmacological interventions (Table 1), it is not surprising to see such a large change in comparison to these published effects. In addition, our sample size was large compared to previous studies, providing more confidence about the results (31). However, because of a lack of a control group and blinding among referrals, we cannot be certain that the outcomes described in this study are solely due to the (psychosocial) interventions applied. These effects may, at least in part, be due to placebo or features such as regression to the mean. Despite that, we accounted for several covariates in our analyses that attempted to minimise these impacts.

In comparison to previous literature (31), our study reported the exact numbers of dementia subtypes in the sample. However, our sample contained a relatively large proportion of unspecified dementia. This is because many referrals may have not received or yet to receive an accurate dementia diagnosis and such diagnosis is not a requirement for DSA service provisions. Further, dementia is underdiagnosed by up to 20% in Australian RACHs where residents may have dementia, but are lacking formal diagnosis (55).

Another limitation is that our analyses did not include the effect of medications. Given the objective of the present study, our aim was to only include the impact of non-pharmacological interventions as they represent the core principle of DSA programs and have been attempted on all referrals. It is worth noting though that each SBRT referral receives a medication review by a psychogeriatrician/geriatrician if they are not already receiving this through external specialists. A medication review is also provided to DBMAS referrals if the DSA consultant identifies the medication(s) may be inappropriately prescribed. Due to the nature of our study design (real-world data with no control group), we cannot rule out the possibility that some or all referrals were provided medications, and that they were supported by other services, and/or simply develop other illnesses (including delirium) or even became more frail during the provision of DSA programs. In the case of de-prescribing, the implementation may be much slower and could extend beyond the length of DSA service provision when compared to the non-pharmacological interventions. Notwithstanding this, future research should address these limitations.

Finally, the outcome data were confined to NPI metrics. The timing of NPI assessments was not the same for all cases, and hence some NPI domain symptoms (e.g., night-time behaviour) may be affected more than others (e.g., agitation) by the fluctuations of diurnal-nocturnal rhythm. However, all consultants received a standardised training on administering NPI assessments in various conditions and were highly experienced in dementia care, somewhat minimising such an effect. Other important data such as clinical (e.g., medication use) and economic (e.g., cost-effectiveness ratio) outcomes will be investigated in future studies.

### Clinical Implications

This study expands upon previous research suggesting that many (if not all) BPSD can be most effectively supported by non-pharmacological interventions. Referrals used DSA services, even in the presence of an acute/severe event, indicating the clinical need of such services. Given the high care costs and resources required for dementia, this service model may play a critical role in overloaded health systems that provide care for affected individuals with multimorbidity and polypharmacy. The importance of demonstrating the benefits of such approaches, in contrast with the small effect sizes and high risks associated with the use of antipsychotics for the management of BPSD, cannot be overstated.

### Conclusions

DSA programs provide a novel, effective and feasible model of care and service delivery specifically designed to address perceived gaps in the aged care system. Our findings add to the body of evidence supporting the efficacy of multidisciplinary and multimodal non-pharmacological strategies in the support of BPSD in aged care residents with various dementia subtypes, and demonstrate that it is possible to implement a highly effective, holistic, and sustainable behaviour support service on a national scale.

## Data Availability Statement

All data used in this article will remain confidential to comply with the conditions of service provision of Dementia Support Australia (DSA).

## Ethics Statement

The study received ethical approval with a waiver of informed consent from the University of New South Wales (UNSW) Human Research Ethics Committee (HC190049). Data handling procedures complied with the World Medical Association's Declaration of Helsinki of ethical principles, and the National Health and Medical Research Council's National Statement on Ethical Conduct in Human Research (2018).

## Author Contributions

SM, MAt, TM, DW, and MH: drafted the first version of the manuscript. SM and MAt: conducted the literature review. DW: processed the data and performed statistical analyses. MAt, TM, and DW: acquisition, analysis, or interpretation of data. SM, MAt, TM, and DW: preparation of manuscript. All authors: study concept and design and critical revision of the manuscript.

## Conflict of Interest

The authors are staff members of The Dementia Centre; a research, education, and consultancy arm of HammondCare, an independent Christian charity which auspices the DSA programs – DBMAS and SBRT. SM is the Head of Clinical Services for The Dementia Centre, HammondCare. MAl is the Head of Business Development, HammondCare. CC is the Director of The Dementia Centre, HammondCare.
